# A large travel-associated outbreak of iatrogenic botulism in four European countries following intragastric botulinum neurotoxin injections for weight reduction, Türkiye, February to March 2023

**DOI:** 10.2807/1560-7917.ES.2023.28.23.2300203

**Published:** 2023-06-08

**Authors:** Martin Bernhard Dorner, Hendrik Wilking, Martin Skiba, Laura Wilk, Maximilian Steinberg, Sylvia Worbs, Sabahat Çeken, Sedat Kaygusuz, Stéphanie Simon, François Becher, Agata Mikolajewska, Christian Kornschober, Timo Bütler, Nathalie Jourdan-Da-Silva, Maria an der Heiden, Lars Schaade, Klaus Stark, Brigitte Gertrud Dorner, Christina Frank

**Affiliations:** 1 Biological Toxins (ZBS3), Centre for Biological Threats and Special Pathogens, Robert Koch Institute, Berlin, Germany; 2 Department for Infectious Disease Epidemiology, Robert Koch Institute, Berlin, Germany; 3 General Directorate of Public Health, Ministry of Health, Ankara, Türkiye; 4 Université Paris Saclay, Commissariat à l’énergie atomique et aux énergies alternatives (CEA), Institut national de recherche pour l'agriculture, l'alimentation et l'environnement (INRAE), Département Médicaments et Technologies pour la Santé (DMTS), SPI, Gif-sur-Yvette, France; 5 Strategy and Incident Response (ZBS7), Centre for Biological Threats and Special Pathogens, Robert Koch Institute, Berlin, Germany; 6 Austrian Agency for Health and Food Safety (AGES), Vienna, Austria; 7 National International Health Regulation (IHR) Focal Point for Switzerland, Swiss Federal Office of Public Health, Division of Communicable Diseases, Bern, Switzerland; 8 Santé Publique France, Saint-Maurice, France; 9 Centre for Biological Threats and Special Pathogens, Robert Koch Institute, Berlin, Germany; * These authors contributed equally to the work and share the last authorship

**Keywords:** Botulism, botulinum neurotoxin, iatrogenic, outbreak, Europe

## Abstract

In March 2023, 34 associated cases of iatrogenic botulism were detected in Germany (30 cases), Switzerland (two cases), Austria (one case), and France (one case). An alert was rapidly disseminated via European Union networks and communication platforms (Food- and Waterborne Diseases and Zoonoses Network, EpiPulse, Early Warning and Response System) and the International Health Regulation mechanism; the outbreak was investigated in a European collaboration. We traced sources of the botulism outbreak to treatment of weight loss in Türkiye, involving intragastric injections of botulinum neurotoxin. Cases were traced using a list of patients who had received this treatment. Laboratory investigations of the first 12 German cases confirmed nine cases. The application of innovative and highly sensitive endopeptidase assays was necessary to detect minute traces of botulinum neurotoxin in patient sera. The botulism notification requirement for physicians was essential to detect this outbreak in Germany. The surveillance case definition of botulism should be revisited and inclusion of cases of iatrogenic botulism should be considered as these cases might lack standard laboratory confirmation yet warrant public health action. Any potential risks associated with the use of botulinum neurotoxins in medical procedures need to be carefully balanced with the expected benefits of the procedure.

**Key public health message**

**What did you want to address in this study?**

Botulism is a potentially life-threatening condition caused by botulinum neurotoxins produced by *Clostridium botulinum* and other clostridia. Apart from the classically occurring forms (food-borne, infant, and wound botulism), the medical and cosmetic application of the neurotoxins gave rise to rare cases of iatrogenic botulism. Here we describe the detection and investigation of an unusual and large multi-country outbreak of iatrogenic botulism.

**What have we learnt from this study?**

We found that 34 persons developed symptoms of botulism after treatment with botulinum neurotoxin injections. Adults from at least Germany, Switzerland, Austria and France had travelled to Türkiye for this treatment for intended weight loss and became ill within days. Several persons were hospitalised and some were ill for weeks.

**What are the implications of your findings for public health?**

Injections with botulinum neurotoxin triggered the disease. The patients received higher doses than those usually used, the reason for this remains unclear. Any potential risks of the medical and cosmetic use of botulinum neurotoxin need to be carefully assessed. This outbreak highlights the cross-border implications of travel-associated illnesses and the importance of surveillance systems to detect unusual and rare diseases.

## Background

Botulism is a flaccid descending paralysis caused by the botulinum neurotoxins (BoNTs) produced by *Clostridium* species. Between 2012 and 2021, on average of 94 cases of human botulism were annually notified in European Union (EU) countries [1]. The neurotoxins can be divided into eight serotypes, A to H; serotypes A, B, E, and F are commonly reported to cause botulism in humans. Serotypes occur in a number of subtypes or mosaic forms (e.g. A1 to A8), which can differ up to 36% at amino acid level [2]. Each serotype can cleave a distinct amino acid bond in the synaptic proteins synaptosomal-associated protein, 25kDa (SNAP-25), vesicle-associated membrane protein (VAMP) and/or syntaxin, essential components of the soluble *N*-ethylmaleimide-sensitive fusion protein (NSF) attachment receptor (SNARE) complex, thereby, preventing formation of the SNARE complex. Formation of the SNARE-complex is an essential step in the fusion of neurotransmitter-loaded vesicle with the synaptic membrane. Consequently, cleavage of SNARE-complex proteins by BoNT prevents neurotransmitter release and leads to paralysis of the innervated muscle [3]. Despite differences in the amino acid sequence of the target proteins and cleavage activities, the resulting clinical picture is similar for all toxin serotypes. Hallmarks of botulism are the paralysis of the facial muscles leading to e.g. ptosis, diplopia, blurred vision, dysarthria and dysphagia. Paralysis can descend causing hypotonia of the extremities and dyspnoea. The clinical picture can worsen over a couple of days and become life-threatening if untreated. Intensive care and mechanical ventilation are used to treat symptoms, and botulism antitoxins can prevent further uptake of BoNTs at the motoneurons [4].

The BoNTs are produced by seven species of the genus *Clostridium: C. botulinum* Group I to Group IV, *C. baratii*, *C. butyricum* and *C. sporogenes*. Each species has different growth habitats and conditions and shows differences in the (heat) resistance of their spores [5]. Botulism is classically divided in three forms; the most common in Europe is food-borne botulism, an intoxication induced by preformed BoNTs in foods. In contrast, infant botulism results from bacterial colonisation of the immature infant intestine and toxin release, while wound botulism is a wound infection.

Botulinum neurotoxin (BoNT)/A- and BoNT/B-containing preparations are available for licensed pharmaceutical use (various brand names and companies) to treat a growing number of neurological and non-neurological diseases including blepharospasm and other facial dystonias, migraine or depression [6,7]. The therapeutic success and excellent safety record paved the way for BoNT pharmaceuticals into other licenced (e.g. cosmetic) or non-licenced (off-label) use. Among the BoNT-containing drugs, BoNT/A1 preparations are most frequently used because of their several months-long half-life [8]. Even if all products contain BoNT/A1 as an active component, not all companies have licenced their product for the treatment of a specific condition. Also, product composition with respect to the presence of BoNT complex proteins and excipients will vary from brand to brand. The stated potency in units is strongly dependent on the assay used and differs between producers. Consequently, the units are not interchangeable, and each product is applied in a product-specific dosage regime which differs between brands [9,10]. For the treatment of blepharospasm, 25–80 units per eye depending on the brand are recommended [11], doses can surge to 600–1,500 units (depending on the brand) for lower extremity spasticity [9,12]. Even though the use of BoNT/A preparation to treat obese patients has not been licenced for any product, several clinics offer intragastric injection with BoNT/A with 100–500 units per treatment [13,14]. Due to the minute amounts of BoNT injected, iatrogenic botulism (IB) is usually observed only in isolated cases often affecting individual nerves and/or muscles adjacent to the injection site with mild to moderate symptoms [8].

## Outbreak detection

On 3 March 2023, the German National Consultant Laboratory (CL) for neurotoxin-producing clostridia at the Robert Koch Institute (RKI) in Berlin, received a call from a German hospital regarding an apparent clinical case of botulism in a patient, who had received intragastric botulinum neurotoxin injections (IBNI) during a gastroscopic procedure in Istanbul, Türkiye, in late February 2023. When additional cases were reported, both the Turkish and the German public health authorities were alerted. European Union and other countries with access to EpiPulse, the European Centre for Disease Prevention and Control (ECDC) and the World Health Organization Regional Office for Europe (WHO/Europe) were informed via a post on the ECDC EpiPulse for the Food- and Waterborne Diseases and Zoonoses Network (FWD Net), an online portal for public health authorities in the EU and partner organisations in additional countries to share information on infectious disease threats. Furthermore, Austria and Germany shared outbreak information via the Early Warning and Response System (EWRS) to other EU member states. In response, Austria, France and Switzerland informed about similar cases. The national Turkish and German International Health Regulations (IHR) Focal Points shared relevant information according to the IHR throughout the investigation. Subsequently, both Türkiye and other European countries reported more cases constituting a large and international outbreak of iatrogenic botulism in Europe in at least five countries.

We here present the relevant epidemiological and laboratory data on travel-associated cases of the first European outbreak of IB which is, to our knowledge, the largest IB outbreak ever described.

## Methods

### Setting and case definitions

Data on the German cases were collected by the CL and through the notification system. Suspected or confirmed botulism in humans is mandatorily notifiable by clinicians, and laboratory-confirmed botulism by laboratories to local health departments, who collect additional data and report the case to the state authorities, who report it to the national level at RKI. The German case definition considers as confirmed not only laboratory-confirmed cases but also purely clinical cases who share the same exposure as a laboratory-confirmed case (epidemiologically linked).

In Austria, France and Switzerland, cases of clinical botulism without a laboratory confirmation are not considered as confirmed.

In Austria, only cases of food-borne botulism are mandatorily notifiable by clinicians and laboratories. Although cases of wound or iatrogenic botulism are not notifiable, the National Reference Centre, the only laboratory performing botulism diagnosis in Austria, is encouraged to inform the health authorities. In France, clinical botulism is notifiable to the regional health authorities who forward the information to Santé Publique France. In Switzerland, botulism is notifiable by clinicians and laboratories except for infant and wound botulism.

Within the EU and European Economic Area (EEA), countries need to notify cases of botulism to the ECDC.

For the purpose of this outbreak investigation and in line with the European case definition, any case of clinical botulism with an exposure history of IBNI in Türkiye was considered as a probable case, cases with additional laboratory evidence of BoNT-intoxication were considered as confirmed.

To actively find cases, we contacted on 22 and 23 March 2023 persons treated with IBNI and with German passports or phone numbers from a list supplied by the Turkish authorities.

### Laboratory methods

The CL requested the earliest possible serum and faecal samples from the suspected cases. Stool samples were cultured anaerobically, the plates were examined for BoNT-producing *Clostridium* species, and a quantitative PCR for BoNT gene cluster genes was performed [15,16]. Serum samples were analysed by mouse bioassay (MBA) by injecting 450 µL of serum intraperitoneally and monitoring the animals for signs of paralysis over 4 days [17].

Under optimal conditions, the MBA can detect around one median lethal dose (LD_50_) which converts to around 4–7 pg (or 8–14 pg/mL) of BoNT/A1 [18]. The potency of BoNT/A-containing drugs is expressed in units, which are based on the mouse median lethal dose, even though units are not interchangeable between brands. Considering the substantial dilution of the applied units (often 1,500 units in this outbreak) by the total blood volume, only sublethal/subclinical amounts (for a mouse) per mL could be expected in the current outbreak. Consequently, more sensitive methods were needed to demonstrate BoNT/A in samples of the current outbreak. To date, only endopeptidase assays have been shown to surpass the high sensitivity of the MBA in the detection of BoNTs in clinical samples [17]. Endopeptidase assays detect the catalytic activity of BoNT’s light chain (LC) based on cleavage of their substrates (SNAP-25, VAMP and/or syntaxin). Each type of LC hydrolyses its substrate at a unique position which leads to the occurrence of defined cleavage products. These products can be detected e.g. by mass spectrometry in the Endopep-MS assay developed at the United States Centers for Disease Control in Atlanta [19,20]. Alternatively, the newly generated N- or C-terminus of the products can be detected by neoepitope-specific antibodies recognising the new epitopes of the N- or C-terminus but showing no reactivity to the uncleaved substrate protein [21-23]. To increase sensitivity and to remove detrimental matrix components for both Endopep-approaches, the BoNT is extracted in a first step by immuno-affinity enrichment using monoclonal antibodies of high affinity. In the Endopep-MS method, the captured BoNT is then incubated with a peptide substrate mimicking the natural substrate protein. Finally, the reaction is analysed by mass spectrometry for the occurrence of the specific cleavage products. For the Endopep-MS, detection limits down to 0.05 LD_50_ in human serum have been reported [24]. In the endopeptidase-suspension immuno-assay (Endopep-SIA) targeting BoNT/A and B simultaneously, BoNT is enriched by monoclonal antibodies and the immuno-captured BoNT/A and BoNT/B is incubated with their natural substrate proteins (human SNAP-25 or VAMP-2) covalently coupled to colour-coded microbeads. To detect proteolytic cleavage events, biotinylated neoepitope-specific antibodies recognising either the cleavage product of BoNT/A in SNAP-25 or the cleavage product of BoNT/B in VAMP-2 were incubated with the SNAP-25 and VAMP-2 beads. Binding of the neoepitope-specific antibodies to the cleaved SNAP-25 or VAMP-2 is visualised by the addition of fluorescently labelled streptavidin which binds to the biotinylated antibodies in a Luminex instrument (Luminex Corporation, Austin, US) [23].

We were able to detect below 10 pg/mL of BoNT/A1 from human serum by the Endopep-SIA and ca 0.1–1 pg/mL by the Endopep-MS assay. A more comprehensive description of the method can be found in the Supplement. Due to the differences in the enrichment of antibodies which recognise different epitopes on BoNT/A, the use of peptides (Endopep-MS) or recombinant SNAP-25 or VAMP-2 proteins (Endopep-SIA) and differences in cleavage buffers used and, more importantly, the independent read-outs (mass-to-charge ratios vs neoepitope recognition), we consider both methods as orthogonal methods.

## Results

By 3 April 2023, 34 botulism cases were reported: nine confirmed and 21 probable cases in Germany, two probable cases in Switzerland, and one probable case each in Austria and France. Thirty-one case patients reported having received IBNI before developing botulism. Three patients claimed to have acquired food-borne botulism in Istanbul and not having received gastric BoNT injection, but their names were on the list of IBNI-treated persons with a residence in Germany supplied by the Turkish authorities. A total of 32 cases were treated in a single private hospital (Hospital X) in Istanbul, Türkiye. One case patient was treated in Istanbul but the hospital name is unknown, and one had IBNI in a private hospital in Izmir (Hospital Y). All treatments were for intended weight loss; one patient also had a stomach balloon inserted.

**Figure 1:**
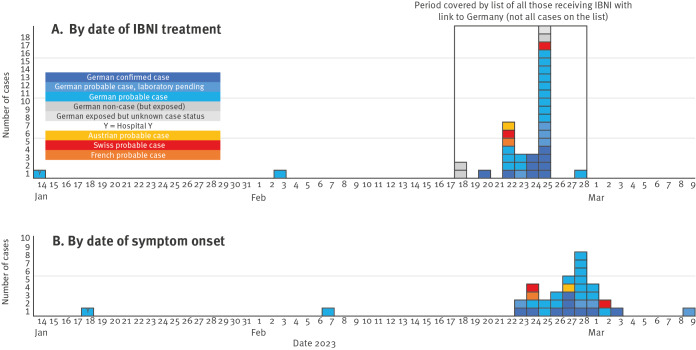
Treatment dates of German, Austrian, French and Swiss case patients with intragastric botulinum neurotoxin injections and dates of onset of symptoms of botulism cases, 14 January–9 March 2023 (n = 34)

The case patients received IBNI once, between 14 January and 28 February 2023 (Figure 1) and reported having received 1,000–2,500 units BoNT/A, with some recalling one and the same brand (BoNT/A product D of brand E). According to the medical records of all case patients, 1,500 units of the product were injected. One case patient stated having received the treatment multiple times before, with no prior adverse effects.

IBNI: intragastric botulinum neurotoxin injections.

Of the case patients, nine were men and 25 were women, with an age range of 20–52 years (median: 36.5). Most (27 of 30) were Turkish or had a background from Türkiye. Body mass index was known for 14 cases and ranged from 25.9 through 39.2 (median: 35) before IBNI. The case patients developed symptoms between 18 January and 9 March 2023, thus 0–12 days after IBNI (median: 3 days); 26 of the 34 cases were subsequently hospitalised with botulism for 2–22 days (median: 7 days). One case patient who fell ill in January was hospitalised three times due to IB, three other case patients were hospitalised twice. Seven of the 32 case patients were treated in intensive care units. Information on antitoxin treatment was available for 24 cases: three of them received botulinum antitoxin. No fatalities were reported.

### Active case finding

In the active case finding, the Turkish authorities provided a list with names of 26 German IBNI patients to the German authorities. Of these, 13 were already known as botulism cases and one was considered a non-case because of very few symptoms and was neither treated with botulinum antitoxin nor hospitalised. Nine of the 11 other cases contacted by local health departments had symptoms of botulism at home; among those, two had an early IBNI treatment date (18 February) and showed no signs of botulism. One individual could not be contacted.

In conclusion, 22 of the 23 individuals treated with INBI after 18 February developed botulism. A retrospective analysis on the clinical picture and disease course is not finalised, but many typical features of botulism were mentioned in the medical reports, including general weakness, dyspnoea, dysarthria, dysphagia, dry mouth, visual impairment including double vision, gait problems, tetraparesis, vomiting, gastroparesis and obstipation.

### Laboratory investigations

**Figure 2:**
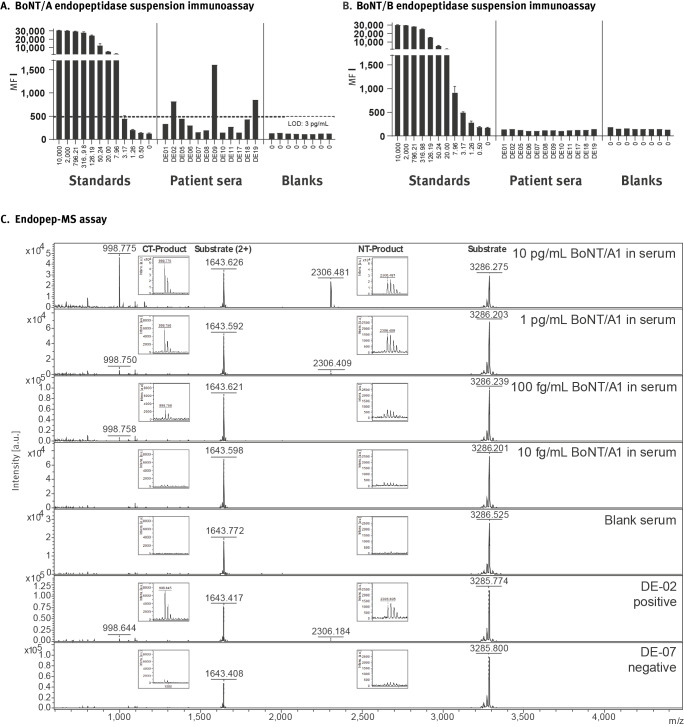
Detection of botulinum neurotoxin A by endopeptidase assays in sera of iatrogenic botulism cases, Germany, February–March 2023 (n = 12)

Results of the CL investigations for the first 12 IB cases notified in Germany are shown in the Table and in Figure 2. Faecal samples that were available from five cases were negative for the presence of BoNT-producing clostridia, thereby excluding food-borne botulism. The MBA results were negative for all 12 case patients. However, BoNT/A, but as expected not BoNT/B, (Figure 1) could be detected in three patients' sera in approximate concentrations between 3 and 8 pg/mL, and in sera from two other cases (DE05, DE18) with borderline positive results using an Endopep-SIA [23].

**Table:** Diagnostic assay results of the first botulism cases notified, Germany, February–March 2023 (n = 12)

Case number	Date of IBNI in 2023	Stool results	Serum				
			Sampling date	MBA	Endopep-SIA	Endopep-MS	Overall result
DE01	25 Feb	NA	2 Mar	−	−	+	+
DE02	25 Feb	−	3 Mar	−	+	+	+
DE05	24 Feb	−	4 Mar	−	( + )	+	+
DE06	24 Feb	−	5 Mar	−	−	+	+
DE07	22 Feb	NA	7 Mar	−	−	−	−
DE08	23 Feb	NA	7 Mar	−	−	−	−
DE09	24 Feb	−	7 Mar	−	+	+	+
DE10	22 Feb	NA	NA	−	−	−	−
DE11	22 Feb	NA	NA	−	−	+	+
DE17	24 Feb	NA	6 Mar	−	−	+	+
DE18	24 Feb	NA	6 Mar	−	( + )	+	+
DE19	24 Feb	−	3 Mar	−	+	+	+

Endopep-MS: endopeptidase mass spectrometry assay; Endopep-SIA: endopeptidase-suspension immunoassay; IBNI: intragastric botulinum neurotoxin injections; MBA: mouse bioassay; NA: not available; +: positive result; ( + ): borderline; − : negative result.

Stool samples were analysed by anaerobic culture and quantitative PCR. For method details, see the Supplement.

a.u.: arbitrary units; BoNT: botulinum neurotoxin; CT: C-terminal; DE: Germany; LOD: limit of detection (in buffer); *m/z*: mass to charge ratio; MALDI-TOF: MALDI-TOF; MFI: median fluorescence intensity; MS: mass spectrometry; NT: N-terminal; SNAP-25: synaptosomal-associated protein of 25kDa; VAMP: vesicle-associated membrane protein.

Panels A and B**: **For toxin enrichment, paramagnetic beads coupled with monoclonal antibodies directed against BoNT/A or BoNT/B were added to either buffer spiked with 10,000 to 0.5 pg/mL toxin as reference (left panel), 12 patients’ sera obtained between 3 and 7 March 2023 (middle panel), or blanks (right panel). After enrichment, the samples were incubated overnight with two different Luminex bead sets coupled with either SNAP-25 or VAMP-2. For the detection of cleaved SNAP-25 or VAMP-2, the Luminex beads were incubated with neoepitope-specific monoclonal antibodies directed against the specific cleavage sites of BoNT/A or BoNT/B. The median fluorescence-intensity (MFI) correlates with the amount of cleaved substrate and therefore indicates the concentration of BoNT in the sample.

Panel C: Human serum was spiked with 10 pg/mL to 10 fg/mL BoNT/A (top four panels) or left untreated (fifth panel). Representative positive or negative patient sera are shown (two bottom panels). For toxin enrichment, the samples were incubated with magnetic beads coupled with an anti-BoNT/A monoclonal antibody. After affinity enrichment, immunocaptured toxins were incubated with a peptide substrate mimic of SNAP-25 overnight. Resulting peptide cleavage products were detected by MALDI-TOF MS with *m/z* = 3,286 representing the intact substrate (*m/z* = 1,643 double-charged substrate peak), *m/z* = 2,306 representing the N-terminal and *m/z* = 998 the C-terminal cleavage product. For method details, see the Supplement.

In addition, an optimised Endopep-MS assay [25], which was based on other antibodies for the immuno-enrichment step, delivered positive results in nine of the 12 cases (Figure 2C) confirming by an orthogonal technique the five positive cases by the Endopep-SIA (see above). We were able to detect below 10 pg/mL of BoNT/A1 from human serum by the Endopep-SIA and ca 0.1–1 pg/mL by the Endopep-MS assay. A more detailed description of both endopeptidase methods is provided in the Supplement. In summary, nine of the first 12 German cases were laboratory-confirmed. However, the sensitivity of the current assays is not sufficient to demonstrate the minute amounts of BoNT, especially if the serum samples are taken late. Thus, negative laboratory results do not rule out the diagnosis if the clinical picture or epidemiological evidence support it.

## Outbreak control measures

In early March, the Turkish authorities closed Hospital X and confiscated the BoNT preparations found. The Turkish Medicines and Medical Devices Agency investigated the material and concluded that a genuine product was found, albeit used in off-label fashion [26,27]. The ECDC and WHO/Europe conducted an international video conference with participants from Türkiye, several EU countries and Switzerland to discuss the outbreak. The outbreak was also featured repeatedly in the Weekly Communicable Disease Threats Report produced by ECDC [28-30] and in the weekly ‘Epidemiologisches Bulletin’ of the RKI [31,32]. In Germany, IBNI patients from the lists provided by Turkish authorities were actively contacted and recommended to see medical care, if they had symptoms typical for botulism. In parallel, additional yet undiagnosed patients were encouraged to seek medical care in both German and Turkish language on Twitter on 10 and 23 March 2023.

## Discussion

By 24 March 2023, 53 cases of apparent IB had been reported in Türkiye between 28 February and 8 March, with 31 hospitalisations. All of these were linked to IBNI conducted by the same physicians in Hospital X (50 cases) and another Hospital Z (three cases); Hospital Y was not mentioned by cases within Türkiye [33]. The BoNT product seized upon discovery of the outbreak, product D of brand E, was approved for import but used off-label. Investigations are still ongoing [26].

The temporal and spatial association of the IBNI treatments with botulism symptoms in the patients outlines them as cases in an outbreak of IB. While MBA was negative in all cases analysed, which was not unexpected due to the time lag and the very low doses of BoNT/A administered, endopeptidase assays with increased sensitivity gave positive results for several cases, confirming the BoNT-intoxication in these patients. This demonstrated the usefulness of endopeptidase assays as animal replacement methods in diagnosis of botulism [17,34] and further supported the designation of the clinical cases as botulism cases belonging to the outbreak. Interestingly, BoNT/A could be detected in patient sera up to 11 days after administration suggesting that BoNT/A was still circulating in the blood in minuscule amounts. It is noteworthy that this outbreak investigation is one of the very few examples where IB could be laboratory-confirmed [35].

Some of the IB cases were only identified by active case finding in Germany, indicating that more persons may have been affected than identified through physician reporting. If patients did not point out their treatment history, general practitioners may have been less likely to suspect botulism when patients presented with milder early symptoms.

Judging from the two early cases identified in Germany, who were both ill for more than 10 weeks after IBNI, the clinical courses appear to be protracted. Compared with other serotypes, BoNT/A excites the longest paralysis times of up to 6 months [36]. Most of the cases we describe here had not completely recovered by mid-April 2023 and may have required additional hospitalisations.

Nearly all IB cases previously described have been sporadic and frequently involve nerves/muscles in the vicinity of the injection side. Even though all brands have based their potency (units) on the mouse median lethal dose, the units are not interchangeable between brands. The biological potency of BoNT/A preparation is not solely based on the amount of BoNT/A but is also influenced by the content of complex proteins and excipients [8]. The use of other than stated diluents for the reconstitution of the lyophilised products can increase the potency of some products [37]. Consequently, every product has been licenced with a product-specific dosages guidance for the individual applications. Besides licenced products, counterfeit products have reached the market [38], and counterfeit products with of up to five times higher-than-stated potency have been seized [39,40], increasing the risk of unintentional overdoses. Counterfeit BoNT-products were blamed for IB outbreaks in the past [41] and the WHO had issued a warning of a counterfeit BoNT/A product (again product D of brand E) in Türkiye in 2022 [42].

Unusual in this outbreak is that most cases clustered in a single hospital (Hospital X), where they all received the same IBNI treatment, and most of them during only 4 days (22–25 February), which indicates a systematic error in the procedure. The isolated earlier cases in Hospital X and Y could be merely coincidental or suggest that this error was transferred between hospitals. Turkish authorities communicated that a genuine product was found, albeit used in off-label fashion [26,27]. It is unclear which product was used in late February. To our knowledge, none of the brands on the market had licenced their BoNT-preparation for IBNI use; still, several clinical and cosmetic applications are off-label uses [43]. Injection of BoNT for the treatment of obesity has gained popularity within the past 20 years and frequently involves product D of brand E, albeit with 500 units, although clear evidence of the benefits of IBNI is lacking [13,14,44-48].

Considering the otherwise excellent safety record of BoNT products, unintentional overdosing perhaps involving a non-licensed product or a change in the procedure would be plausible explanations in the event described here. This outbreak demonstrates a previously unrecognised potential for large IB outbreaks associated with a procedure using BoNT and there are several lessons to be learnt. The systematic error apparently leading to BoNT overdoses and subsequent botulism in the IBNI patients needs to be elucidated, such that it can be actively avoided in the future. At least in view of the 2022 warning by WHO regarding a counterfeit BoNT/A product in Türkiye [42], regulatory mechanisms to keep counterfeit BoNT products out of circulation may need to be reviewed.

Possible risks associated with BoNT use in medical procedures need to be carefully balanced with the expected benefit of the procedure. Symptoms of botulism should be outlined as potential adverse events in pre-procedure patient information.

The potential sustainable benefit of IBNI needs further investigation, ideally in randomised controlled clinical trials with lengthy follow-up. Especially where patients travel long distances for a treatment and/or where treatments may need to be repeated periodically, the motivation to achieve longer-lasting effects by increasing BoNT dosage is understandable but carries additional risk. Where procedures such as IBNI are carried out during very brief foreign travel, it would be beneficial to require arrangements for care of post-procedural complications in the country of residence. Adult patients with symptoms of botulism should be asked about iatrogenic exposures to BoNT and encouraged to mention such exposures so that additional cases can be prevented.

## Conclusion

Resting botulism surveillance on both laboratory and physician notification requirements, as is the case in Germany and France, has been effective in detecting IB, for which laboratory confirmation is likely to fail by standard laboratory tests. The current outbreak highlights impressively the great promise of modern endopeptidase assays in diagnosing even minute amounts of BoNT remaining in serum. These assays may at some point be able to replace the MBA, provided that comprehensive validation studies become available.

## Supplementary Data

119000 Supplement
