# Supplementary material for: A large travel-associated outbreak of iatrogenic botulism in four European countries following intragastric botulinum neurotoxin injections for weight reduction, Türkiye, February to March 2023
Source: Euro Surveill. 2023 Jun 8;28(23):2300203. doi: 10.2807/1560-7917.ES.2023.28.23.2300203 (PMC10318948; doi:10.2807/1560-7917.ES.2023.28.23.2300203)
Supplement: Supplement [file 23-00203_DORNER_SupplementaryInformation.pdf]

Supplementary information on the laboratory confirmation of botulism and a detailed description of the Endopep-MS and Endopep-SIA used.

This supplementary material is hosted by *Eurosurveillance* as supporting information alongside the article: "A large travel-associated European outbreak of iatrogenic botulism in 3 EU countries and Switzerland following intragastric botulinum neurotoxin injections for weight reduction in Türkiye in early 2023", on behalf of the authors, who remain responsible for the accuracy and appropriateness of the content. The same standards for ethics, copyright, attributions and permissions as for the article apply. Supplements are not edited by *Eurosurveillance* and the journal is not responsible for the maintenance of any links or email addresses provided therein.

Laboratory confirmation of botulism

Diagnostics of botulism is based on either the identification of a BoNT-producing *Clostridium* species from faecal samples (i.e., in foodborne and infant botulism) or wound material (wound botulism) by anaerobic culture and PCR for toxin genes, or alternatively by the detection of BoNT from serum or other clinical matrices. Due to the low concentrations of BoNTs in the circulation the detection is very demanding. Historically, the mouse bioassay (MBA) is used which can detect 10–20 pg of BoNT/A per mL, or 10–100 pg/mL for other serotypes. More recent approaches focus on the detection of the BoNTs' cleavage activity (endopeptidase assays). Here, the BoNTs are affinity purified from a matrix and incubated under defined cleavage conditions with either the natural substrate proteins or mimicking peptide substrates. Cleavage can be detected by i) using fluorescently labelled substrates and Förster-resonance energy transfer (FRET) to monitor cleavage, ii) identifying product peptides on the basis of the mass-to-charge ratio by mass spectrometry (MS) in an Endopep-MS format or iii) the use of neoepitope-specific antibodies to detect the newly generated N- or C-terminal ends in the cleaved substrates in an immunoassay (Endopep suspension immunoassay, Endopep-SIA). Based on the literature, the latter two approaches have the potential to surpass the sensitivity of the MBA and were tested in this outbreak (limit of detection of the Endopep-MS assay 0.1–1 pg/mL and Endopep-SIA 3 pg/mL for BoNT/A, respectively). A more detailed method description can be found below. The endopeptidase assays are promising approaches but require the immunoenrichment of the BoNTs from a clinical matrix. This first step is challenged by the high variability of BoNT sero- and subtypes and thus requires an extensive validation for the highly diverse family of BoNTs (1).

Endopep-suspension immunoassay (Endopep-SIA)

A detailed description of the Endopep-SIA principle is given in von Berg *et al.* (2). For simultaneous extraction of BoNT/A and B from samples, paramagnetic M270 Dynabeads carboxylic acid (Invitrogen, Dreieich, Germany) coupled via EDC/Sulfo-NHS with toxin-specific monoclonal antibodies (mAbs) were used. These mAbs are targeting the respective BoNT Hc-fragments and were generated at the RKI earlier following a method described previously (39. For enrichment of BoNT/A mAb A2807 was used, while a combination of B488 and recombinantly produced B1051 (the latter kindly provided by Maren Krüger and Hans-Werner Mages, RKI) was used for BoNT/B. Extraction bead mixes were prepared by mixing equal volumes of mAb-coupled beads. A 1:1-mixture of recombinant BoNT/A and BoNT/B (Toxogen, Hannover, Germany) in standard buffer (0.1 % (w/v) bovine serum albumin (BSA; Carl Roth, Karlsruhe, Germany) in Dulbecco's phosphate buffered saline (PBS), 136.89 mM NaCl, 2.68 mM KCl, 1.47 mM KH_2HPO_4, 8.1 mM Na_2HPO_4, pH 7.3) was used as toxin standard with dilutions ranging from 10000 pg/mL to 0.5 pg/mL. For immunoenrichment of BoNTs, 15 µL of mAb-coupled beads were added to either 200 µL of toxin standards and blanks (standard buffer) per well in a 96 well microtiter plate (Greiner Bio-One, Frickenhausen, Germany) or to 500 µL of patient sera in screw cap microtubes. Toxin standards and blanks were then incubated with mAb-coupled beads for 1 h at room temperature on a multiplate shaker at 600 rpm. Samples containing patients' sera were incubated at room temperature for 2 h with over-head rotation. The beads were then collected from patients' sera

by transferring samples to a magnetic stand (Invitrogen, Dreieich, Germany), allowing for removal of sera and resuspension of mAb-coupled beads in 200 µL of standard buffer. Bead suspension was then transferred to the 96 well microplate containing both toxin standards and blanks. All beads were then washed 3× with PBS-T (0.1 % (v/v) Tween 20 in PBS) followed by 3 washes with deionized water using a Tecan HydroSpeed magnetic plate washer (Tecan AG). Beads were resuspended in 100 µL per well of cleavage buffer (50 mM HEPES, 250 µM $ZnCl_2$, 1 % (v/v) Tween 20, 0.75 M TMAO, 25 mM DTT, pH 7.0). BoNT substrates SNAP-25 (recombinant human protein, 1–206 aa; NKMAX, formerly ATGen, Seongnam-si, Republic of Korea) and VAMP-2 (recombinant human protein, 1-89 aa; ProSpec-Tany TechnoGene Ltd., Ness-Ziona, Israel) were coupled via EDC/Sulfo-NHS chemistry to distinctly coloured MagPlex Microspheres (Luminex Corporation, Austin, TX, United States). Substrate beads were diluted 1:20 in cleavage buffer of which 25 µL were added per well (final microsphere concentration per well: 10 beads/region/µL). The cleavage reaction was carried out over night for 20 h at 37 °C under constant back and forth movement of the microtiter plate. Following incubation, beads were washed twice with PBS-T. In order to detect substrate cleavage sites, 100 µL of biotinylated neoepitope specific monoclonal antibodies SNAP/A/291 and VAMP/B/1488 (2) in 1 % (w/v) BSA/PBS were added per well, both with a concentration of 2 µg/mL. Samples were incubated at room temperature for 90 min on a multiplate shaker at 600 rpm followed by washing twice with PBS-T. Beads were resuspended in 100 µL per well streptavidin-R-phycoerythrin (Agilent, PJRS34-1, Santa Clara, United States) diluted to 2 µg/mL in 1 % (w/v) BSA/PBS and incubated at room temperature for 30 min on a multiplate shaker at 600 rpm. Finally, beads were washed three times with PBS-T and resuspended in 100 µL 1 % (w/v) BSA/PBS. Sample measurement was performed on a Bio-Plex 200 instrument (Bio-Rad Laboratories GmbH, Munich, Germany) with high RP1 target and doublet discriminator gates set to 8000–22000.

Endopep-MS assay

The Endopep-MS assay was performed according to Wang *et al.* and Kull *et al.* (4,5) with several modifications. As first step, the toxin was immunoaffinity enriched from patients' sera or control samples by an anti-BoNT/A monoclonal antibody (mAb TBA23, Stéphanie Simon, CEA, France) immobilised onto M-280 tosyl-activated paramagnetic Dynabeads (Invitrogen, Dreieich, Germany). Antibody immobilisation was done according the manufacturer's protocol as described earlier (5). 450 µL of a patients' sera were added to 50 µL of 10-fold phosphate buffered saline with 0.1% Tween (PBS-T) buffer and 20 µL of TBA23 antibody-coated beads. As positive controls, different concentrations of BoNT/A1 reference material (generated and qualified in the EQuATox project (6)) were spiked into 450 µL human donor serum resulting in concentrations ranging from 10 fg/mL to 1 ng/mL. As negative control human donor serum without toxin was used. For immunoaffinity enrichment, the toxin was automatically processed in a KingFisher Flex purification system (Thermo Scientific, Vantaa, Finland). Antibody-coated beads were incubated in serum by mixing for 2 h at room temperature, washed twice with 1 mL PBST with 2 M NaCl to remove unspecific bindings (7), washed twice with 1 mL PBST and once with 1 mL and 100 µL of water (LC-MS grade, Carl Roth, Karlsruhe, Germany). Washed beads were reconstituted in 20 µL of Endopep-MS reaction buffer consisting of 20 mM HEPES (pH 7.3), 1 mg/mL bovine serum albumin, 10 mM dithiothreitol, 200 µM $ZnCl_2$ and SNAP-25 peptide substrate Ac-(Nal)(Nal)EKAPARGFNKPKIDAGNGRATR(Nle)LGGR-NH_2 (with Nal: 2-Napthylalanine and Nle: norleucine) (4) at a final concentration of 50 µM. The peptide substrate was synthesized by peptides & elephants (Hennigsdorf, Germany) and delivered at a purity of >95%. Samples were then incubated at 37 °C for 17 h without agitation in a PCR cycler. A 2-µL aliquot of each reaction supernatant was mixed with 18 µL of MALDI matrix solution consisting of α-cyano-4-hydroxy cinnamic acid (Fluka, Buchs, Switzerland) at 5 mg/mL in 50% acetonitrile (Carl Roth, Karlsruhe, Germany), 0.1% TFA and 1 mM ammonium citrate (both Sigma-Aldrich, Seelze, Germany). Then, 1 µL of this mixture was spotted on an MTP 384 polished steel target plate (Bruker Daltonics, Bremen, Germany). Mass spectra of each spot were acquired over the mass range *m/z* 600 to 4500 in MS-positive ion reflector mode on an autoflex speed matrix-assisted laser desorption/ionization time-of-flight (MALDI-TOF) mass spectrometer (Bruker Daltonics, Bremen, Germany) equipped with a smartbeam laser. For matrix suppression, deflection was set to *m/z* 600. External mass calibration was performed with peptide calibration standard II (Bruker Daltonics, Bremen, Germany). Each spectrum represents the sum of 4000 laser shots. Spectra were processed using the flexAnalysis 3.4 software (Bruker Daltonics, Bremen, Germany). Positive

samples were indicated by the cleavage of the peptide substrate (Ac-(Nal)(Nal)EKAPARGFNKPKIDAGNGRATR(Nle)LGGR-NH_2; *m/z* 3286) resulting in the N-terminal (NT) cleavage product at *m/z* 2306 (Ac-(Nal)(Nal)EKAPARGFNKPKIDAGNG) and C-terminal (CT) one at *m/z* 998 (RATR(Nle)LGGR-NH_2).

References

1. Stern D, von Berg L, Skiba M, Dorner MB, Dorner BG. Replacing the mouse bioassay for diagnostics and potency testing of botulinum neurotoxins – progress and challenges. Berl Munch Tierarztl Wochenschr. 2018;131(9/10):375-94.
2. von Berg L, Stern D, Pauly D, Mahrhold S, Weisemann J, Jentsch L, et al. Functional detection of botulinum neurotoxin serotypes A to F by monoclonal neoepitope-specific antibodies and suspension array technology. Sci Rep. 2019;9(1):5531.
3. Pauly D, Kirchner S, Stoermann B, Schreiber T, Kaulfuss S, Schade R, et al. Simultaneous quantification of five bacterial and plant toxins from complex matrices using a multiplexed fluorescent magnetic suspension assay. Analyst. 2009;134(10):2028-39.
4. Wang D, Baudys J, Hoyt KM, Barr JR, Kalb SR. Further optimization of peptide substrate enhanced assay performance for BoNT/A detection by MALDI-TOF mass spectrometry. Anal Bioanal Chem. 2017;409(20):4779-86.
5. Kull S, Pauly D, Störmann B, Kirchner S, Stämmler M, Dorner MB, et al. Multiplex detection of microbial and plant toxins by immunoaffinity enrichment and matrix-assisted laser desorption/ionization mass spectrometry. Anal Chem. 2010;82(7):2916-24.
6. Weisemann J, Krez N, Fiebig U, Worbs S, Skiba M, Endermann T, et al. Generation and characterization of six recombinant botulinum neurotoxins as reference material to serve in an international proficiency test. Toxins (Basel). 2015;7(12):5035-54.
7. Wang D, Baudys J, Kalb SR, Barr JR. Improved detection of botulinum neurotoxin type A in stool by mass spectrometry. Anal Biochem. 2011;412(1):67-73.
